# A Few November Flowers

**Published:** 1886-11-20

**Authors:** 


### "A FEW NOVEMBER FLOWERS."

Mrs. Williams, Rochford House, near Tenbury, Worcestershire, writes to say she will be much obliged if the editor of The Hospital will kindly send her the name and address of the gentleman who wrote poetry in The Hospital of October 30th, called, "A Few November Flowers," as she would so much like to send him some flowers.

*** It is not possible to comply with Mrs. Williams' request, but if the flowers could be sent to Dr. Hack Tuke, 63, Welbeck Street, Cavendish Square, W., by 10 o'clock any Wednesday or Saturday morning, he has kindly undertaken to deliver them to the writer of the poetry in question.—Ed. T. H.

